# Effects of Tirzepatide on Skeletal Muscle Mass in Adults: A Systematic Review

**DOI:** 10.7759/cureus.89020

**Published:** 2025-07-29

**Authors:** Roberto A Hidalgo Ramos, Isaac Hong, Marcelo Ortiz, Daniela Secades, Sebastián Dufner Krieger, Linet Ramos Stanziola

**Affiliations:** 1 Faculty of Medicine, University of Costa Rica, San Jose, CRI; 2 Anesthesiology, Hospital San Francisco de Asis, Grecia, CRI

**Keywords:** body composition, gip, glp-1, lean mass, obesity, skeletal muscle, tirzepatide, type 2 diabetes

## Abstract

Tirzepatide has become a treatment option for weight management, with increasing interest in its effects on skeletal muscle. This systematic review evaluated the impact of tirzepatide on skeletal muscle mass and lean body composition in adults. A structured search of four major databases was conducted up to June 26, 2025, following Preferred Reporting Items for Systematic reviews and Meta-Analyses (PRISMA) guidelines. The included studies were randomized clinical trials that assessed skeletal muscle or lean mass using validated imaging methods in adults treated with tirzepatide. The available evidence suggests that treatment is associated with reductions in fat mass while maintaining the relative preservation of lean mass. Indicators of muscle composition remained stable or showed signs of improvement. These findings support the potential of tirzepatide to improve overall body composition without significantly compromising skeletal muscle. Further research is needed to confirm these results and explore their functional significance over time.

## Introduction and background

The primary incretin hormones responsible for mediating the incretin effect are glucagon-like peptide-1 (GLP-1) and glucose-dependent insulinotropic polypeptide (GIP). GLP-1 promotes glucose-dependent insulin secretion while simultaneously suppressing glucagon release under both normoglycemic and hyperglycemic conditions [1]. Additionally, it contributes to delayed gastric emptying and enhanced satiety [2]. GIP also stimulates insulin secretion in a glucose-dependent manner and accounts for a substantial portion of the incretin response [1]. Interestingly, GIP demonstrates glucagonotropic effects in normoglycemic and hypoglycemic states and glucagonostatic properties during hyperglycemia [1]. Furthermore, GIP enhances insulin sensitivity in adipose tissue and improves the postprandial lipid-buffering capacity of adipose tissue [2].

Tirzepatide is a groundbreaking dual agonist of both GIP and GLP-1 receptors that has gained attention as a pharmacological intervention for obesity. Clinical studies have demonstrated that tirzepatide can achieve substantial weight loss, with reductions approaching 25% of initial body weight over an 18-month period [3]. While the magnitude of weight reduction is clinically significant, it is important to recognize that not all of the weight lost may be attributable to fat mass [4]. This raises concerns regarding potential adverse effects on lean body mass, particularly skeletal muscle, which is metabolically active and plays a critical role in energy expenditure [5]. Preserving muscle mass during weight loss is essential for sustaining metabolic health and improving long-term outcomes [6]. Therefore, elucidating tirzepatide’s impact on lean body mass is crucial for assessing the quality of weight loss and its broader implications for long-term health.

## Review

Methods

Study Scope and PICO Framework

This systematic review focused on studies involving adult human participants, either healthy or with common comorbidities such as obesity, type 2 diabetes, or hypertension. The intervention of interest was the administration of tirzepatide, in any formulation or dosage. While the inclusion of a comparator group was not required, eligible studies could include individuals who were untreated, not receiving antiobesity or antidiabetic medications, or treated with alternative pharmacologic regimens for similar indications. The primary outcomes assessed were changes in skeletal muscle mass, skeletal muscle percentage, or lean mass, measured through validated imaging techniques or body composition analysis methods.

Eligibility and Exclusion Criteria

Studies were eligible for inclusion if they were published within the last 10 years, conducted in adult human populations, written in English, and evaluated skeletal muscle mass, skeletal muscle percentage, or lean mass in participants receiving tirzepatide. Only clinical trials, randomized or nonrandomized, were included. 

Studies conducted in animals, pediatric populations, or individuals with severe illnesses or major comorbidities such as cancer or recent surgical procedures were excluded. Studies involving individuals with type 1 diabetes mellitus were also excluded. Additionally, studies that combined tirzepatide with interventions likely to significantly influence muscle composition, such as other antiobesity medications or bariatric surgery, were excluded if those interventions were a primary focus, treatment strategy, or inclusion criterion. Case reports, systematic reviews, editorials, and studies that were not clinical trials were also excluded.

Search Strategy

A structured search of PubMed, EMBASE, Web of Science, and the Cochrane Library was conducted up to June 26, 2025, following Preferred Reporting Items for Systematic reviews and Meta-Analyses (PRISMA) guidelines. The objective was to identify clinical trials evaluating the effects of tirzepatide on skeletal muscle mass, skeletal muscle percentage, or lean mass in adults.

The search combined controlled vocabulary and free-text terms using Boolean operators. The general query applied was ("Tirzepatide" OR "Mounjaro" OR "Tirzepatida") AND ("Muscle" OR "Muscle Mass" OR "Skeletal Muscle" OR "Lean Mass"). An example of the database-specific syntax is shown for EMBASE: ('tirzepatide'/exp OR tirzepatide:ti,ab,kw OR mounjaro:ti,ab,kw OR tirzepatida:ti,ab,kw) AND ('muscle'/exp OR muscle:ti,ab,kw OR 'muscle mass':ti,ab,kw OR 'skeletal muscle':ti,ab,kw).

No filters were initially applied. After retrieval, studies were screened according to predefined eligibility criteria, and duplicates were removed. References of relevant articles were also reviewed manually to identify any additional eligible trials.

Study Selection, Data Extraction, and Quality Assessment

Two authors (Hong and Ortiz) independently screened the titles and abstracts of all studies identified from the initial search. Any disagreements were resolved by a third investigator (Dufner). Studies that met the preliminary inclusion criteria were selected for full-text review. The same reviewers assessed the full-text articles for eligibility based on predefined criteria, and inclusion was determined by consensus. Data extraction was performed collaboratively by all three reviewers. Given that both included studies were randomized controlled trials, quality assessment was conducted using the Risk of Bias 2 tool (RoB 2, Cochrane Collaboration, London, UK), and this evaluation was reviewed and approved by two independent investigators (Hidalgo and Secades).

Endpoints and Definitions

The primary endpoint of this systematic review was the change in skeletal muscle mass in adults treated with tirzepatide, including both absolute changes and percentage changes from baseline. When studies included additional skeletal muscle mass-related changes, such as lean mass, these were also considered in this review. Lean body mass, or lean mass, refers to total body weight minus the weight of fat and can be measured indirectly using dual-energy X-ray absorptiometry (DXA) or MRI. No other secondary endpoints were included for this review.

Results

Study Selection

**Figure 1:**
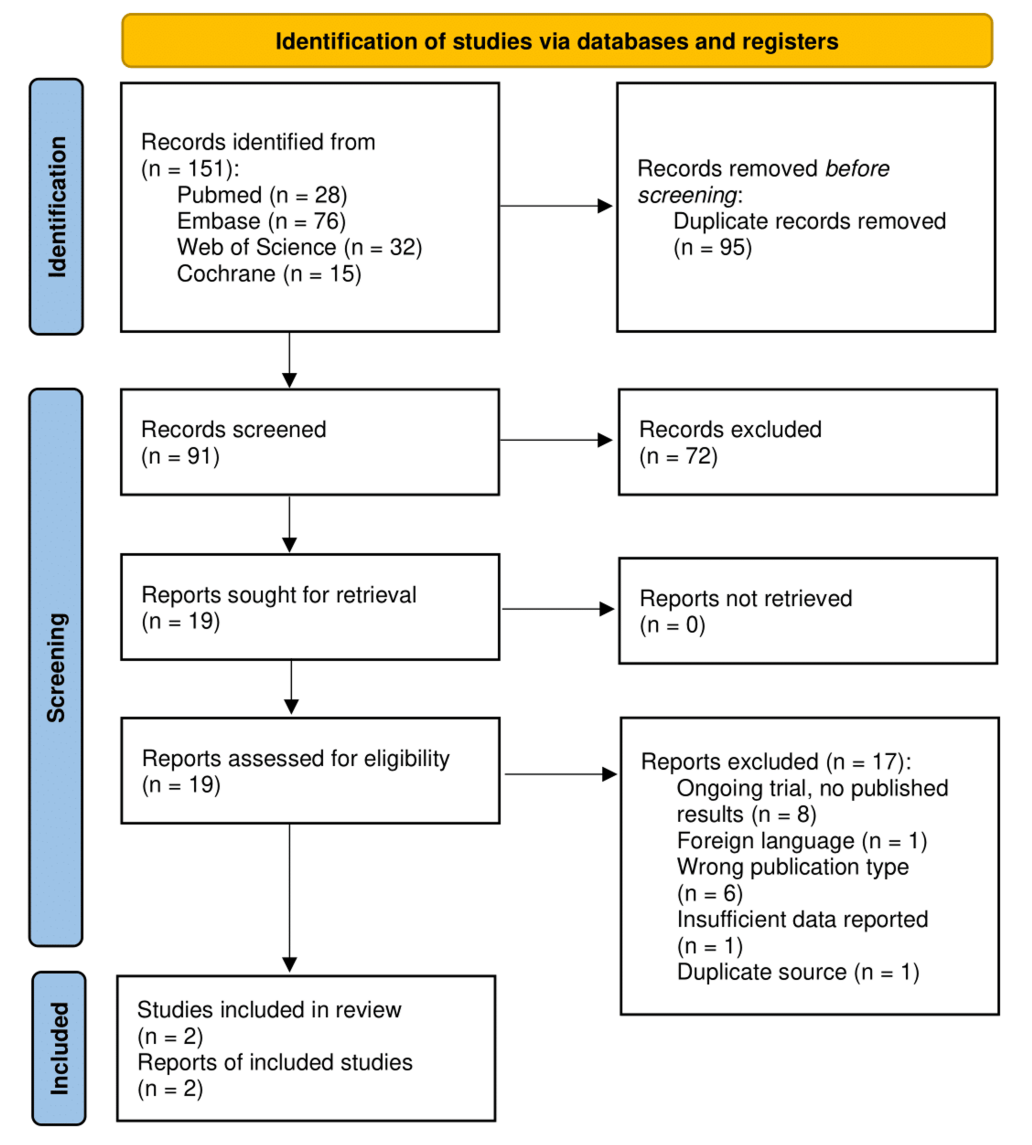
PRISMA flow diagram. PRISMA: Preferred Reporting Items for Systematic reviews and Meta-Analyses.

A total of 151 records were retrieved from PubMed, EMBASE, Web of Science, and Cochrane databases. After removing 95 duplicates, 91 articles were screened by title and abstract, with 19 selected for full-text assessment. Only two studies met the eligibility criteria and were included in the final review. The selection process is illustrated in Figure 1, following the PRISMA flow diagram.

Quality Assessment

The methodological quality of the two post hoc analyses was evaluated using the RoB 2. As shown in Table 1, both studies demonstrated an overall low-to-moderate risk of bias. Although most domains were rated as low risk, there were some concerns regarding the selection of reported outcomes and study design limitations inherent to post hoc analyses, which may introduce bias.

**Table 1:** Risk of bias assessment of included studies (RoB 2). Risk of bias assessment using the RoB 2 tool across five domains: (D1) randomization, (D2) deviations from interventions, (D3) missing data, (D4) outcome measurement, and (D5) selective reporting. RoB 2: Risk of Bias 2 tool.

Study	D1	D2	D3	D4	D5	Overall
Look et al. [7]	Low	Low	Some concerns	Low	Some concerns	Some concerns
Sattar et al. [8]	Low	Low	Low	Low	Some concerns	Some concerns

Characteristics of Included Studies

This review includes two clinical studies that evaluated the effects of tirzepatide on skeletal muscle mass and composition in adult populations: a substudy of the SURMOUNT-1 trial and a substudy of the SURPASS-3 trial (MRI arm). Both were randomized controlled trials sponsored by Eli Lilly and Company, focusing on changes in body composition with tirzepatide treatment in populations with obesity or type 2 diabetes.

In both studies, tirzepatide was administered subcutaneously once weekly, and lifestyle advice was provided, though structured exercise programs were not formally included. The trials differed in follow-up duration, 72 weeks in SURMOUNT-1 and 52 weeks in SURPASS-3 MRI, and in population characteristics, with SURMOUNT-1 including people without diabetes and SURPASS-3 focusing on individuals with type 2 diabetes [7,8].

Table 2 presents detailed characteristics of the included studies to provide an overview of the design and methodological features of the selected trials. These include the study design, population characteristics, sample size, intervention and comparator arms, methods of body composition measurement, as well as lean mass outcomes and statistical significance.

**Table 2:** Summary of study design and participant allocation of included studies. SURPASS-3 MRI used insulin degludec (Novo Nordisk, 100 U/mL, subcutaneous, 3 mL pen) as comparator.

Study ID	Study design	Location	Population characteristics	Sample size	Intervention	Comparator	Muscle measurement method
Look et al. [7]	Substudy of phase 3, multicenter, double-blind placebo-controlled trial	Argentina, Brazil, Mexico, Puerto Rico, Taiwan, and the United States	Adults without type 2 diabetes with a BMI ≥30 kg/m^2^ or ≥27 kg/m^2^ with one or more weight-related complication(s)	124	Pooled tirzepatide 5, 10, and 15 mg	Placebo	Whole-body DXA scanners with body composition capability
Sattar et al. [8]	Randomized, open-label, parallel-group, phase 3 trial	Argentina, Austria, Greece, Hungary, Italy, Romania, Spain, and the United States	Adults with T2D, insulin-naive, BMI ≥25, HbA1c 7.0–10.5%, on metformin ± SGLT2i	190	Participants were randomly assigned to weekly subcutaneous tirzepatide (5, 10, or 15 mg)	Insulin degludec 100 U/mL, once daily SC	Fat-free muscle volume measured by MRI at baseline and at week 52

Summary of Key Findings

The SURMOUNT-1 DXA substudy, reported by Look et al. [7], included 160 adults with overweight or obesity, of whom 124 received tirzepatide (5, 10, or 15 mg once weekly). Participants were predominantly female (73%), with a mean baseline weight of 102.5 kg, BMI of 38.0 kg/m², and average age of 46.2 years. DXA was used at baseline and at 72 weeks to measure body composition, including fat mass and lean mass. Tirzepatide treatment led to a 21.3% mean reduction in body weight, with approximately 75% of the weight loss attributable to fat mass and 25% to lean mass. The preservation of lean mass proportions was consistent across subgroups stratified by age, sex, and degree of weight loss.

The SURPASS-3 MRI substudy, reported by Sattar et al. [8], focused specifically on skeletal muscle quality in 296 adults with type 2 diabetes and a fatty liver index ≥60. Participants were randomized to tirzepatide (5, 10, or 15 mg) or insulin degludec and followed for 52 weeks. MRI was used to quantify muscle fat infiltration (MFI) and fat-free muscle volume (FFMV). Tirzepatide significantly reduced MFI across all doses compared to insulin degludec, indicating improved muscle composition. Importantly, reductions in fat-free muscle volume were small and within expected ranges based on reference data from the UK Biobank, suggesting tirzepatide does not cause clinically significant skeletal muscle loss.

Together, these studies provide evidence that tirzepatide induces significant fat mass loss while preserving a relatively stable proportion of lean mass and improving markers of muscle composition, such as MFI, particularly in adults with obesity or type 2 diabetes. A side-by-side comparison of both studies' results is shown in Table 3, which highlights the absolute and percentage changes in lean mass observed with tirzepatide treatment.

**Table 3:** Effects of tirzepatide on lean mass and muscle composition in included studies. This table summarizes outcomes from the included trials on changes in lean mass and muscle composition with tirzepatide, using DXA (SURMOUNT-1) and MRI (SURPASS-3 MRI) over 72 and 52 weeks, respectively. DXA: dual-energy X-ray absorptiometry; ETD: estimated treatment difference; TZD: tirzepatide.

Study ID	Intervention	Mean absolute change in lean mass was	Lean mass (percent change)	Follow-up duration for LDL	Statistical significance reported
Look et al. [7]	Pooled TZD 5, 10, and 15 mg	-5.6	-10.9%	72 weeks	Mean change in lean mass: ETD: -8.3 [95% CI: -10.6, -6.1] p<0.001). Mean absolute change in lean mass: ETD: -4.4 [95% CI: -5.6, -3.2] p<0.001)
Sattar et al. [8]	Participants were randomly assigned (1:1:1:1) to receive TZD (5 mg, 10 mg, or 15 mg), once per week as a subcutaneous injection	-0.6 L female -0.7 L male	-6.9% female -5.5% male	52 weeks	p=0.22, general p<0.0001 (TZP 15 mg)

Discussion

This systematic review evaluated the effects of tirzepatide on skeletal muscle mass and overall body composition in adults. Evidence from two randomized controlled trials suggests that tirzepatide promotes substantial weight loss primarily through reductions in fat mass while preserving lean mass and improving muscle composition.

The SURMOUNT 1 DXA substudy provided detailed longitudinal data on individuals with overweight or obesity treated over a 72-week period [7]. Approximately 75% of the weight loss was attributable to fat mass, while only 25% was from lean mass, demonstrating a favorable preservation of metabolically active tissue. These results were consistent across demographic subgroups, including age, sex, and degree of weight loss, highlighting the broad applicability of these findings.

In parallel, the SURPASS 3 MRI substudy focused on skeletal muscle quality in adults with type 2 diabetes [8]. Tirzepatide treatment led to significant reductions in muscle fat infiltration and preserved fat-free muscle volume within clinically acceptable ranges over 52 weeks, suggesting not only the preservation of skeletal muscle quantity but also improvements in muscle quality. These findings were reinforced by preliminary data presented at the European Association for the Study of Diabetes Annual Meeting, which reported consistent reductions in muscle fat infiltration across all tirzepatide doses.

Preserving muscle mass during weight loss is clinically important because the loss of lean mass, particularly in older adults or individuals with comorbidities, has been associated with reduced mobility, increased frailty, and impaired metabolic health. As emphasized by Wilkinson et al. [9], lean body mass preservation plays a vital role in maintaining functional capacity and overall physical resilience. Similarly, Lucas and Aronne [10] advocated for redefining obesity by incorporating body composition metrics, which is especially relevant when evaluating interventions like tirzepatide that demonstrate selective fat mass reduction.

This review has important limitations to consider. Both included studies were conducted in collaboration with Eli Lilly, the manufacturer of tirzepatide, which may introduce potential bias despite rigorous methodologies and transparent reporting. Additionally, one study was a post hoc analysis, raising concerns about selective reporting. The small number of included studies and their relatively short follow-up periods further limit the generalizability of the findings. Independent, long-term studies are needed to validate these results and ensure their applicability to broader populations.

In summary, the current evidence supports tirzepatide as a pharmacologic agent capable of achieving significant fat mass reduction while preserving skeletal muscle mass and improving muscle composition. These results are particularly encouraging in the context of managing obesity and type 2 diabetes, where maintaining muscle health is critical to long-term metabolic and functional outcomes. Continued investigation is needed to assess durability, functional performance, and generalizability across diverse populations.

## Conclusions

This systematic review highlights that the studies reviewed reported significant weight loss in adults with obesity and/or type 2 diabetes following treatment with tirzepatide, while also preserving skeletal muscle mass and improving muscle composition. The SURMOUNT-1 DXA substudy demonstrated that approximately 75% of the weight loss was attributable to fat mass, with only 25% from lean mass. Likewise, the SURPASS-3 MRI substudy showed reductions in muscle fat infiltration and stable fat-free muscle volume, suggesting preservation of muscle quantity and enhancement of muscle quality. These findings support tirzepatide as a promising pharmacologic option for managing obesity and metabolic disease without compromising skeletal muscle health. Further long-term studies are warranted to confirm these outcomes and to evaluate their implications for muscle strength, physical performance, and long-term functional health.
